# Huge Gastrointestinal Stromal Tumor (GIST) in Upper Gastrointestinal Masquerade Anaemia

**DOI:** 10.7759/cureus.62409

**Published:** 2024-06-14

**Authors:** Muhammad Syamiel Irfan Zahidin, Jetinder Singh, Sumayyah Mohammad Azmi, Azzahra Azhar, Mohd Nizam Md Hashim, Andee Dzulkarnaen Zakaria

**Affiliations:** 1 Department of Surgery, School of Medical Sciences, Universiti Sains Malaysia, Kota Bharu, MYS; 2 Department of Pathology, School of Medical Sciences, Universiti Sains Malaysia, Kota Bharu, MYS; 3 Department of Pathology, Universiti Sultan Zainal Abidin, Kampus Gong Badak, Kuala Terengganu, MYS; 4 Department of Colorectal Surgery, Hospital Universiti Sains Malaysia, Kota Bharu, MYS

**Keywords:** mesenchymal tumour, upper gastrointestinal(ugi) bleeding, anaemia, gastrointestinal (gi), gastrointestinal stromal tumour (gist)

## Abstract

Gastrointestinal stromal tumor (GIST) represents a rare neoplasm affecting the gastrointestinal (GI) tract and is classified as a common nonepithelial tumor within the GI tract. It originates from the interstitial cells of Cajal, and GIST typically manifests with symptoms such as abdominal pain, weight loss, and gastrointestinal bleeding. This case involves a 33-year-old male who presented with GI bleeding symptoms after eight months of treatment for anemia. Oesophagogastroduodenoscopy (OGDS) revealed a singular ulcerated mass measuring 4x4cm while a computed tomography (CT) scan identified a large fundal exophytic component extending from the gastroesophageal junction to the stomach. Subsequently, the patient underwent a laparotomy and proximal gastrectomy with Roux-en-Y reconstruction, which revealed a 12x10 cm tumor located at the fundus of the stomach. This report aims to underscore the potential for misdiagnosis in the initial presentation of GIST, emphasizing the importance of raising clinical awareness in such cases.

## Introduction

Gastrointestinal stromal tumor (GIST) makes up less than 1% of all gastrointestinal tumors, although it predominates among mesenchymal tumors in the alimentary canal [1]. At first, GIST was mislabelled as schwannoma, leiomyoma, and leiomyosarcoma. As the condition has progressed, developments in ultrastructural, immunohistochemical, and molecular biology techniques have made it easier to determine that GIST is derived from common progenitor cells or interstitial cells of Cajal (ICC) [2]. GISTs can appear in any part of the GI system, although the stomach and small intestine are more common sites for them to appear. Less than 5% of GISTs are extra-GI GISTs, which originate within the abdominal cavity and have no direct link to the GI tract [3].

## Case presentation

**Figure 1:**
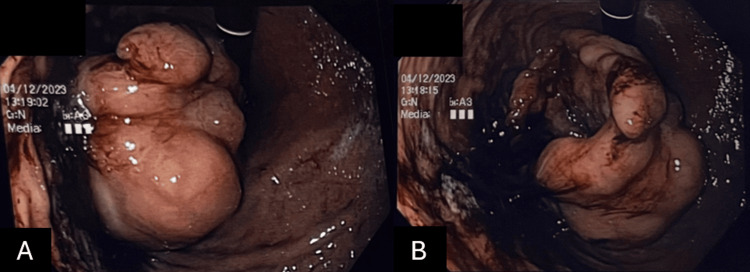
A&B: Oesophagogastroduodenoscopy (OGDS) showing a single ulcerated mass measuring 4x4 cm

**Figure 2:**
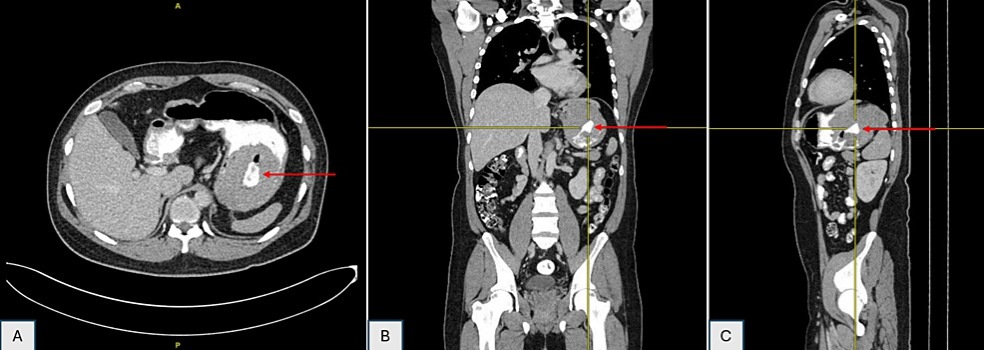
Exophytic component noted posteriorly on the CT thorax, abdomen, and pelvis A - axial view; B - coronal view, C - sagittal view

A 33-year-old male with no known medical illness presented with a pre-syncopal attack, lethargy, and intermittent palpitation for five months. He had lost 12 kg over 8 months. The patient also complained of early satiety for one month, before presenting with coffee-ground vomitus, melena stool, and epigastric discomfort for three days. On abdominal examination, the abdomen appeared to be soft and non-tender, with no palpable mass, and no distension noted. An oesophagogastroduodenoscopy (OGDS) was done to identify the cause of the bleeding. The OGDS revealed a single ulcerated mass measuring 4x4 cm. Altered blood in the stomach was noted and no other ulcer or mass was noted (Figure 1). A CT scan revealed an ulcerated ulcer measuring 4x4 cm at the gastroesophageal junction, with an exophytic component with a small area of necrosis at the posterior and the superior part (Figure 2).

**Figure 3:**
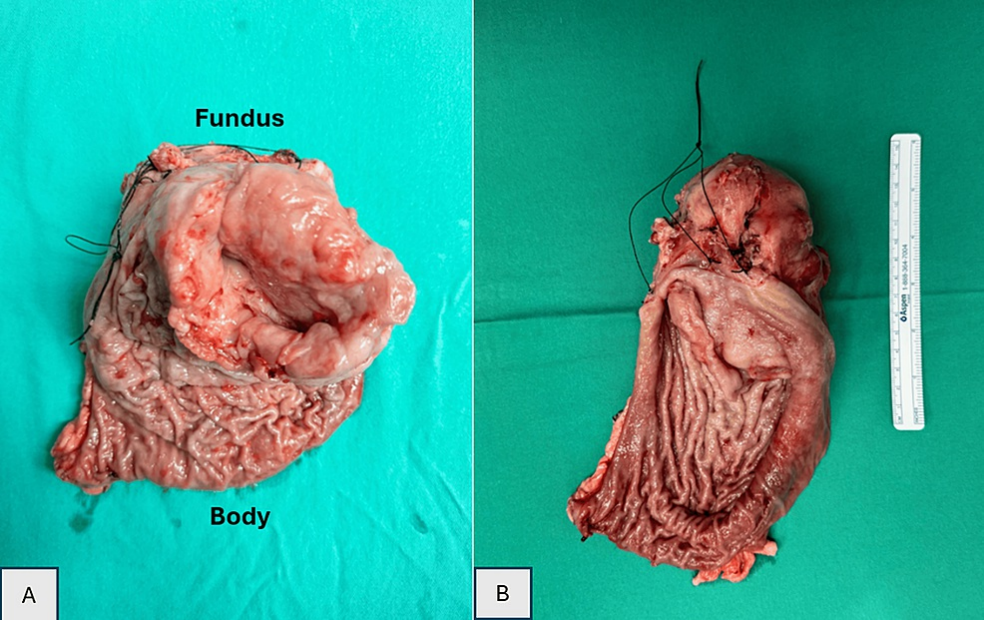
A: A well-defined, firm mass arising from the muscularis propria layer, with erosion of the mucosal surface; B: A well-defined mass, with soft to firm consistency, measuring 12X10 cm, with no perforation seen

On open proximal gastrectomy, a large tumor was seen at the fundus of the stomach measuring about 12x10 cm, with no adhesions or infiltrations to the surrounding organs. The serosa of the stomach was intact, and the tumor was submucosal, with signs of rupture into the gastric cavity. However, no blood was noted in the stomach. Roux-en-Y reconstruction and oesophagojejunal anastomosis were proceeded with (Figure 3).

**Figure 4:**
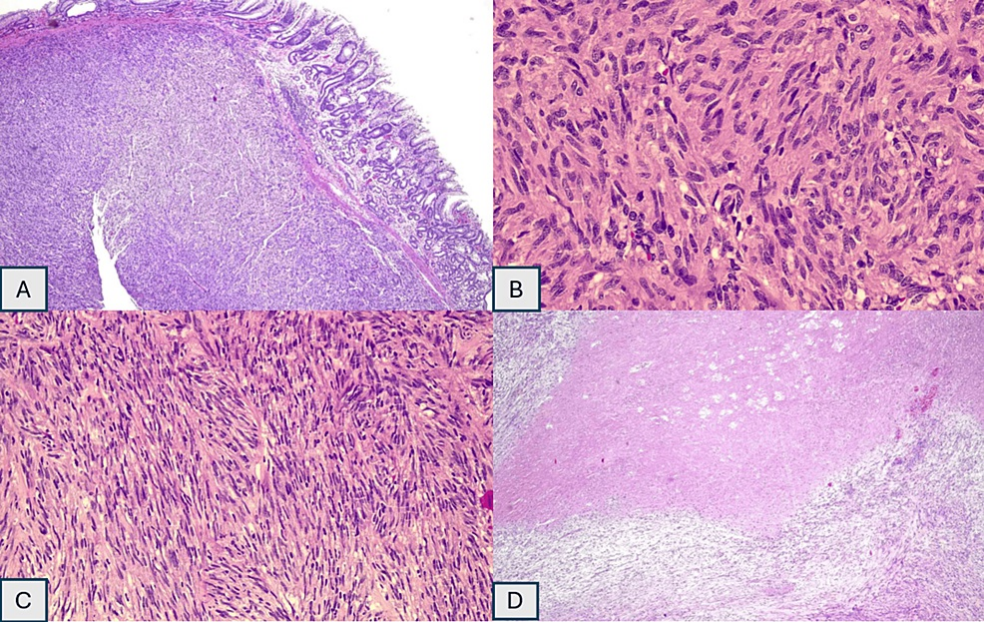
A: 4x; section shows a well-circumscribed tumor arising from within the muscularis propria composed of predominant, fairly monotonous, spindle-shaped cells. B: 100X; these spindle-shaped cells have elongated to slender nuclei, inconspicuous nucleoli, and abundant pale eosinophilic cytoplasm. Mitoses seen in 10HPF. C: 200X; paranuclear vacuolization is apparent. D: 4X; foci of necrosis present.

**Figure 5:**
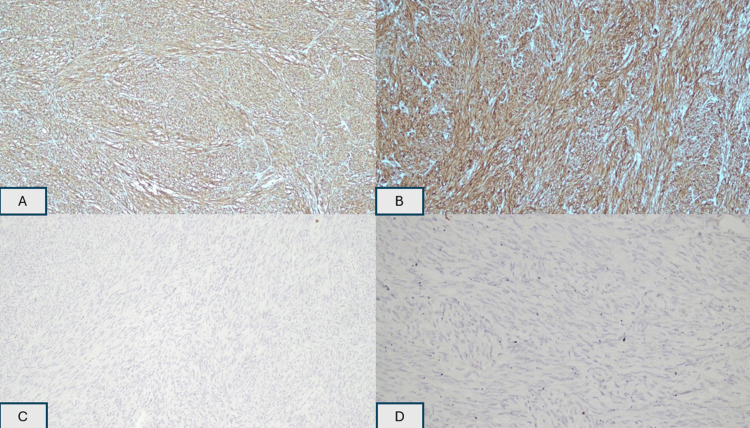
These spindle-shaped tumor cells are strong and diffusely positive toward CD34 and CD117 and negative towards S100 and SMA immunostain. A: CD34; B: CD117; C: S100; D: smooth muscle actin (SMA) CD: cluster of differentiation

The histopathological examination revealed a well-circumscribed tumor arising from within the muscularis propria and consisting of predominant, fairly monotonous, spindle-shaped cells (Figure 4). The tumor cells are positive toward CD34 and CD117 and negative toward S100 and SMA immunostain (Figure 5).

## Discussion

Mesenchymal neoplasms that arise in the GI tract are known as GISTs, and they are usually KIT-positive. The autonomic nervous gastrointestinal system's pacemaker cells, the interstitial cells of Cajal (ICC), and the stem cells that are linked to them are thought to be the source of these tumors [4].

Genetic variety is seen in GISTs; the most common primary drivers have been found to be mutations related to KIT, PDGFRA, and SDHx. On the other hand, less common drivers are mutations in the RAS pathway (KRAS, BRAF, NF1). Different tumor biologies and responses to tyrosine kinase inhibitor (TKI) therapy are influenced by the wide range of mutations. In particular, GISTs expressing KIT mutations demonstrate responsiveness to TKIs while GISTs lacking KIT mutations show resistance to this treatment modality [5]. The average age at which GISTs appear is 59.7 years, and despite some research [6-8] suggesting an equal sex occurrence, there is a significant male predominance. There are ethnic differences in the frequency of the condition; the Chinese population is most susceptible, followed by Malays, Indians, and other ethnic groups [9].

Gastrointestinal bleeding is the most common sign of GISTs, and other symptoms include weight loss, dysphagia, abdominal pain, and the presence of an abdominal mass [10]. In this particular case, anemia symptoms were first observed, with a hemoglobin level of 4.4 g/dl. However, no obvious signs or symptoms of gastrointestinal bleeding were seen.

Macroscopically, GISTs are usually described as tan to white lesions with well-defined margins within the gastric walls. These tumors may manifest as a variety of histologic subtypes, but the most common ones are spindle cell type, epithelioid cell type, and mixed spindle-epithelioid type. Different histologic subtypes of the spindle cell type include sarcomatous spindle cell, palisading-vacuolated subtype, hypercellular subtype, and sclerosing spindle cell. The subtypes of the epithelioid cell type include sarcomatous epithelioid GISTs, dyscohesive epithelioid, hypercellular epithelioid, and sclerosing epithelioid variation [11]. Histopathology revealed the most common subtype, the spindle cell type.

Surgical intervention is the principal treatment modality for localized GISTs, with the aim of achieving total removal. To ensure a sufficient surgical margin, this requires removing the tumor along with its pseudo capsule. Surgical methods include laparoscopic and endoscopic cooperative surgery, as well as enucleation, submucosal dissection, submucosal excavation, band ligation, full-thickness resection, and submucosal tunneling endoscopic resection [12].

Four subtypes of GISTs are distinguished according to their location in relation to the muscularis propria.

Type I: Tumor protruding into the digestive lumen with a narrow connection to the muscularis propria.

Type II: Tumor protruding into the digestive lumen with a wide connection to the muscularis propria.

Type III: Tumor centrally localized on the gastric wall.

Type IV: Tumor protruding into the serosa of the gastric wall.

Safe endoscopic resections can be an excellent option for gastric GISTs that are smaller than 4 cm in size. On the other hand, there is a higher chance of recurrence and possible metastasis when the diameters are greater than 4 cm. In certain situations, tyrosine kinase inhibitor adjuvant therapy may be required or a combination of endoscopic and surgical methods may be used [12].

After surgically removing GISTs, there is a greater chance of peritoneal metastasis due to the independent risk factor of tumor rupture. In the absence of adjuvant treatment, patients with a ruptured GIST in the abdominal cavity had a recurrence probability of approximately 94% (15 out of 16; 93.7%). Fifteen out of 23 cases had an overall risk of peritoneal metastasis of 65.2%; 9 of those cases involved the peritoneum alone, and 6 cases included both the peritoneum and liver. Interestingly, few published studies specifically address the rates of metastasis and recurrence associated with endoscopic resection for ruptured GISTs [13].

The final diagnosis for this patient was a ruptured fundal GIST, which is characterized by a large tumor, 10 × 12 cm in size, which is located at the fundus of the stomach. This measurement is marginally larger than the normal range of 5-8 cm observed in these types of tumors [14]. The tumor was determined to be submucosal and showed clear indications of rupture into the gastric cavity. Tumor resection was the appropriate choice of action in this patient's care. As a result, elective surgery - open proximal gastrectomy with Roux-en-Y reconstruction - was the preferred approach. The goal of this surgery was to manage and mitigate the effects of the ruptured GIST that was found in the stomach's fundus.

## Conclusions

Understanding the various etiological factors that could lead to anemia is vital for medical professionals, considering the possibility of neoplastic origins. When there is gastrointestinal bleeding, the onset of anemia symptoms emphasizes the importance of comprehensive investigations. An immediate and thorough examination, including imaging studies such as endoscopy and CT, may be needed to rule out GI causes. Late detection may lead to worse progression such as rupture, thus increasing the risk of metastasis. In this case, the patient had a ruptured GIST and underwent surgical intervention with no intraoperative and postoperative complications observed.

## References

[1] Enodien B, Hendie D, Müller T, Taha-Mehlitz S, Frey DM, Taha A (2023). Gastrointestinal stromal tumor (GIST): a remarkable case report and literature review. Cureus.

[2] Ramzi A, Perwita AD, Salsabila D, Putri KT, Azmi U (2023). Gastrointestinal stromal tumor (GIST). J Soc Sci.

[3] von Mehren M, Joensuu H (2018). Gastrointestinal stromal tumors. J Clin Oncol.

[4] Caterino S, Lorenzon L, Petrucciani N (2011). Gastrointestinal stromal tumors: correlation between symptoms at presentation, tumor location and prognostic factors in 47 consecutive patients. World J Surg Oncol.

[5] Sharma AK, de la Torre J, IJzerman NS (2021). Location of gastrointestinal stromal tumor (GIST) in the stomach predicts tumor mutation profile and drug sensitivity. Clin Cancer Res.

[6] Miettinen M, Sarlomo-Rikala M, Lasota J (1999). Gastrointestinal stromal tumors: recent advances in understanding of their biology. Hum Pathol.

[7] Khoo JJ, Gunn A (2005). A clinical and immunohistochemical study of gastrointestinal stromal tumours. Malays J Pathol.

[8] Steigen SE, Bjerkehagen B, Haugland HK (2008). Diagnostic and prognostic markers for gastrointestinal stromal tumors in Norway. Mod Pathol.

[9] Krishnappa P, Loh EJ, Mohammed IB, Tata MD, Akhilesh M, Palayan K (2016). Histomorphology and immunohistochemistry of gastrointestinal stromal tumors in a Malaysian population. Asian Pac J Cancer Prev.

[10] Scarpa M, Bertin M, Ruffolo C, Polese L, D'Amico DF, Angriman I (2008). A systematic review on the clinical diagnosis of gastrointestinal stromal tumors. J Surg Oncol.

[11] Tan CB, Zhi W, Shahzad G, Mustacchia P (2012). Gastrointestinal stromal tumors: a review of case reports, diagnosis, treatment, and future directions. ISRN Gastroenterol.

[12] Gheorghe G, Bacalbasa N, Ceobanu G (2021). Gastrointestinal stromal tumors-a mini review. J Pers Med.

[13] Song S, Ren W, Wang Y (2018). Tumor rupture of gastric gastrointestinal stromal tumors during endoscopic resection: a risk factor for peritoneal metastasis?. Endosc Int Open.

[14] Ortiz WJ, Landazuri-Navas S, Moron-Cabrera N (2022). Gastric antral gastrointestinal stromal tumor presenting with severe anemia. Cureus.

